# Association between unmet social needs and healthy lifestyle parenting behaviors

**DOI:** 10.3389/fped.2023.1015610

**Published:** 2023-02-23

**Authors:** Kelsey A. Egan, Man Luo, Meghan Perkins, Ines Castro, Megan Sandel, Caroline J. Kistin, Elsie M. Taveras, Lauren Fiechtner

**Affiliations:** ^1^ Division of General Academic Pediatrics, Department of Pediatrics, Boston Medical Center, Boston University School of Medicine, Boston, MA, United States; ^2^ Division of General Pediatrics, Department of Pediatrics, MassGeneral Hospital for Children, Boston, MA, United States; ^3^ Department of Nutrition, Harvard T.H. Chan School of Public Health, Boston, MA, United States; ^4^ Division of Gastroenterology and Nutrition, MassGeneral Hospital for Children, Boston, MA, United States; ^5^ The Greater Boston Food Bank, Boston, MA, United States

**Keywords:** food insecurity, housing insecurity, childhood obesity, parenting behaviors, nutrition, physical activity

## Abstract

**Objective:**

To examine cross-sectional associations of food and housing security risks and healthy lifestyle parenting behaviors related to nutrition and physical activity among families with children with overweight/obesity.

**Methods:**

We surveyed 407 parents of children ages 6–12 years with overweight/obesity. Exposures were measures of food and housing insecurity risk. Outcomes were healthy lifestyle parenting behaviors related to nutrition and physical activity. Logistic regression models for each exposure-outcome relationship were adjusted for parental educational attainment, parental cohabitation status, household size, and household income.

**Results:**

In multivariable-adjusted models, food insecurity was associated with significantly lower odds of parent modeling exercise {aOR 0.60 [95% confidence interval (CI): 0.37, 0.96]} and parent modeling eating healthy foods [aOR 0.42 (95% CI: 0.24, 0.73)]. Housing insecurity was associated with significantly lower odds of parent modeling exercise [aOR 0.57 (95% CI: 0.35, 0.95)].

**Conclusions:**

Food insecurity and housing insecurity may be barriers to parents adopting and modeling healthy lifestyle parenting behaviors related to physical activity and nutrition.

## Introduction

Socio-economic and racial/ethnic disparities in childhood obesity have persisted (1, 2). In order to reduce disparities in childhood obesity, we must better understand the barriers and competing demands that families face, such as food and housing insecurity, and how these unmet social needs may impact healthy lifestyle behaviors.

Parents play an important role in promoting healthy lifestyle behaviors for their children by modeling eating healthy foods and exercise, and providing access to healthy food and physical activity opportunities. Studies have found a positive relationship between nutrition and physical activity-related parenting behaviors and children's healthy lifestyle behaviors (3, 4). Additionally, pediatric weight management interventions that incorporate parent participation have been found to be more effective (5). Given the critical role of parents in shaping healthy lifestyle behaviors of their children, we need to better understand whether stressors, such as food and housing insecurity, could make it more difficult for parents to carry out these healthy parenting behaviors.

Little is known about the impact of unmet social needs on healthy lifestyle parenting behaviors. While a few studies have examined associations of food insecurity with feeding practices of caregivers, predominantly assessed in infancy (6–9), no studies examining the impact of housing insecurity on healthy lifestyle parenting behaviors could be identified. The objective of this study was to examine the association between food and housing insecurity and healthy lifestyle parenting behaviors in a sample of predominantly low-income Hispanic families who have children with overweight or obesity. We hypothesized that food insecurity would be associated with parenting behaviors related to nutrition and that housing insecurity would be associated with parenting behaviors related to nutrition and physical activity.

## Methods

We conducted a cross-sectional analysis of baseline data from the Clinic and Community Approaches to Healthy Weight trial, a two-arm randomized trial in two communities in Massachusetts with predominantly Hispanic, low-income populations. The study was conducted from 2017 to 2019 and aimed to compare the effects of two pediatric weight management interventions. Children were referred by primary care clinicians at participating federally qualified health centers and were eligible if they were ages 6 to 12 with a body mass index ≥ 85th percentile and had a parent that spoke English or Spanish. Children were ineligible if they had a sibling enrolled in the study, had a medical condition affecting their growth or physical activity, did not have a parent or guardian that could follow study procedures for 1 year, or their family was planning on leaving the health center within the study time frame. Primary care providers referred children to the study by electronic referral or electronic fax during a health care visit and a study coordinator subsequently contacted families by phone to obtain informed consent. Additional details regarding study design are published elsewhere (10). A total of 407 child-parent pairs were enrolled in the study, completed the baseline survey, and were included in this analysis. Baseline data used in this analysis were collected through a 20-minute phone survey with the child's parent or guardian, which was conducted prior to randomization assignment or distribution of intervention materials. The Massachusetts Department of Public Health Institutional Review Board approved the study.

Exposures were measures of food and housing insecurity. Risk of food insecurity was assessed using the validated 2-item Hunger Vital Sign (11):

1. Within the past 12 months we worried whether our food would run out before we got money to buy more.
2. Within the past 12 months the food we bought just didn't last and we didn't have money to get more.

Response options included: often true, sometimes true, or never true. An affirmative response (often true or sometimes true) to either question was categorized as food insecure.

Risk of housing insecurity was assessed with questions from the National Survey of America's Families 2002 Questionnaire (12):

1. During the last 12 months, was there a time when you and your family were not able to pay your mortgage, rent or utility bills?
2. During the last 12 months, did you or your children move in with other people even for a little while because you could not afford to pay your mortgage, rent or utility bills?

Response options were yes/no. An affirmative response to either question was categorized as housing insecure.

Outcomes were assessed with individual items drawn from validated measures of parental support for physical activity (Activity Support Scale for Multiple Groups; ACTS-MG) (13) and parental feeding practices (Comprehensive Feeding Practices Questionnaire; CFPQ) (14). Each item was rated with a 4-point Likert scale (strongly agree = 4, agree = 3, disagree = 2, strongly disagree = 1), modified slightly from the Likert scale in the CFPQ to maintain consistency among items. These Likert scales were converted into a dichotomous agree (strongly agree or agree) or disagree (disagree or strongly disagree) for the purposes of this analysis:

1. I exercise or am physically active on a regular basis (13).
2. I take my child to places where he/she can be active (13).
3. I model healthy eating for my child by eating healthy foods myself (14).
4. Most of the food I keep in the house is healthy (14).

Descriptive statistics were used to characterize the overall sample stratified by exposures. A separate logistic regression model was created for each exposure-outcome combination. We included *a priori* confounders in adjusted models that have strong theoretical associations with the exposures and outcomes and have been controlled for in similar studies in the literature (7, 8, 15, 16). Confounders included: parental educational attainment, parental cohabitation status, household size, and household income. The household income variable was dichotomized with a cutoff of $20,000, which is approximately 100% of the federal poverty level for a family of 4 in 2017 (17). Individuals with missing data were excluded from the adjusted models. A significance level of *α* = 0.05 was used for all statistical tests. Data were analyzed using SAS version 9.4.

## Results

Descriptive statistics of 407 parent and child participant pairs are presented in Table 1. Overall, 69% of families reported an income of $20,000 or less and 93% of children were Hispanic. Forty nine percent of families screened positive for food insecurity and 30% screened positive for housing insecurity.

**Table 1:** Sample characteristics in the Clinic and Community Approaches to Healthy Weight trial by food and housing security status (*N* = 407).

	Overall *N* = 407 (100%)	Food insecure *N* = 200 (49%)	Food secure *N* = 207 (51%)	Housing insecure *N* = 121 (30%)	Housing secure *N* = 286 (70%)
	***N* (%) or mean (SD)**				
**Parental/family demographics**					
**Parental educational attainment** ^a^					
Some high school or less	177 (44%)	87 (44%)	90 (44%)	49 (40%)	128 (45%)
High school degree	132 (33%)	67 (34%)	65 (32%)	35 (29%)	97 (34%)
Some college or higher	94 (23%)	43 (22%)	51 (25%)	37 (31%)	57 (20%)
**Parental cohabitation status**					
Cohabitating	152 (37%)	74 (37%)	78 (38%)	41 (34%)	111 (39%)
Not cohabitating	255 (63%)	126 (63%)	129 (62%)	80 (66%)	175 (61%)
**Household size (# of people)**	4.2 (1.3)	4.0 (1.1)	4.3 (1.4)	4.0 (1.1)	4.3 (1.3)
**Household income** ^b^					
$20,000 or less	218 (69%)	109 (75%)	109 (63%)	75 (77%)	143 (65%)
More than $20,000	100 (31%)	37 (25%)	63 (37%)	22 (23%)	78 (35%)
**Parental country of birth** ^c^					
United States	261 (64%)	125 (62.5%)	136 (66%)	82 (68%)	179 (63%)
Other	145 (36%)	75 (37.5%)	70 (34%)	39 (32%)	106 (37%)
**Child demographics**					
**Age (years)**	9.6 (1.8)	9.5 (1.8)	9.7 (1.9)	9.4 (1.8)	9.7 (1.8)
**Gender (female)**	183 (45%)	90 (45%)	93 (45%)	52 (43%)	131 (46%)
**Race/ethnicity** ^c^					
Hispanic	377 (93%)	181 (91%)	196 (95%)	109 (90%)	268 (94%)
Non-Hispanic Black	19 (5%)	12 (6%)	7 (3%)	9 (7%)	10 (4%)
Non-Hispanic White	9 (2%)	6 (3%)	3 (1%)	3 (2%)	6 (2%)
Non-Hispanic Asian	1 (0.2%)	0 (0%)	1 (0.5%)	0 (0%)	1 (0.4%)
**Healthy lifestyle parenting behaviors** ^d^					
Parent modeling exercise	250 (61%)	114 (57%)	136 (66%)	65 (54%)	185 (65%)
Takes child to locations for physical activity^c^	332 (82%)	162 (81%)	170 (83%)	100 (83%)	232 (81%)
Parent modeling eating healthy foods^c^	318 (78%)	143 (72%)	175 (85%)	87 (72%)	231 (81%)
Mostly healthy foods at home	315 (77%)	148 (74%)	167 (81%)	89 (74%)	226 (79%)

^a^ Missing = 4.

^b^ Missing = 89.

^c^ Missing = 1.

^d^ Parent agrees or strongly agrees.

In the unadjusted model (Table 2), food insecurity was not statistically significantly associated with parent modeling exercise {OR 0.69 [95% confidence interval (CI): 0.46, 1.03]}, whereas after adjusting for confounders, the confidence interval narrowed slightly and the association was statistically significant [aOR 0.60 (95% CI: 0.37, 0.96)]. In the unadjusted model, food insecurity was associated with lower odds of parent modeling eating healthy foods [OR 0.44 (CI: 0.27, 0.73)] and the association remained significant in the fully-adjusted model [aOR 0.42 (95% CI: 0.24, 0.73)]. In the partially-adjusted model (not adjusted for income), food insecurity was associated with keeping mostly healthy foods at home [aOR 0.59 (95% CI: 0.36, 0.96)] and after adjusting for income the effect estimate was similar with a slightly widened confidence interval [aOR 0.61 (95% CI: 0.36, 1.04)]. In unadjusted and fully-adjusted models, there were no significant associations between food insecurity and taking child to locations for physical activity.

**Table 2:** Logistic regression models for association between food and housing security status and healthy lifestyle parenting behaviors (*N* = 407).

	**Parent modeling exercise**		
	M1	M2	M3
	OR [95% CI]		
Food insecure	0.69 (0.46, 1.03)	0.72 (0.48, 1.09)	**0.60 (0.37, 0.96)**
Food secure	Ref (1)	Ref (1)	Ref (1)
Housing insecure	**0.63 (0.41, 0.98)**	**0.64 (0.41, 1.00)**	**0.57 (0.35, 0.95)**
Housing secure	Ref (1)	Ref (1)	Ref (1)
	**Takes child to locations for physical activity** ^a^		
	M1	M2	M3
	OR [95% CI]		
Food insecure	0.90 (0.55, 1.50)	0.84 (0.50, 1.40)	0.94 (0.53, 1.66)
Food secure	Ref (1)	Ref (1)	Ref (1)
Housing Insecure	1.09 (0.62, 1.90)	1.12 (0.64, 1.98)	1.02 (0.55, 1.89)
Housing secure	Ref (1)	Ref (1)	Ref (1)
	**Parent modeling eating healthy foods** ^a^		
	M1	M2	M3
	OR [95% CI]		
Food insecure	**0.44 (0.27, 0.73)**	**0.40 (0.24, 0.68)**	**0.42 (0.24, 0.73)**
Food secure	Ref (1)	Ref (1)	Ref (1)
Housing insecure	**0.60 (0.37, 0.98)**	0.63 (0.38, 1.04)	0.65 (0.37, 1.15)
Housing secure	Ref (1)	Ref (1)	Ref (1)
	**Mostly healthy foods at home**		
	M1	M2	M3
	OR [95% CI]		
Food insecure	0.68 (0.43, 1.09)	**0.59 (0.36, 0.96)**	0.61 (0.36, 1.04)
Food secure	Ref (1)	Ref (1)	Ref (1)
Housing insecure	0.74 (0.45, 1.21)	0.75 (0.45, 1.25)	0.74 (0.43, 1.30)
Housing secure	Ref (1)	Ref (1)	Ref (1)

M1 = Unadjusted.

M2 = Adjusted for parental educational attainment (missing = 4), parental cohabitation status, and household size.

M3 = Adjusted for parental educational attainment (missing = 4), parental cohabitation status, household size, and income (missing = 89).

^a^ Missing = 1.

In the unadjusted model (Table 2), housing insecurity was significantly associated with lower odds of parent modeling exercise [OR 0.63 (95% CI: 0.41, 0.98)] and this association remained significant in the fully-adjusted model [aOR 0.57 (95% CI: 0.35, 0.95)]. In the unadjusted model, housing insecurity was associated with lower odds of parent modeling eating healthy foods [OR 0.60 (95% CI: 0.37, 0.98)], but this association was no longer significant in the fully-adjusted model [aOR 0.65 (95% CI: 0.37, 1.15)]. In unadjusted and fully-adjusted models, there were no significant associations between housing insecurity and taking child to locations for physical activity or keeping mostly healthy foods at home.

## Discussion

In this study of predominantly low-income Hispanic children with overweight or obesity, food insecurity was associated with lower odds of parental modeling of exercise and parental modeling of eating healthy foods and housing insecurity was associated with lower odds of parental modeling of exercise, after adjustment for confounders.

Our study's finding that food insecurity was associated with healthy lifestyle parenting behaviors related to nutrition is consistent with previous literature demonstrating associations between food insecurity and feeding practices, predominantly in infants and toddlers (6–9). A potential mechanism to explain our study's food insecurity findings may be that families without stable availability of, and access to, a quality food supply might not have the resources to model healthy eating.

Our finding that food and housing insecurity were associated with lower odds of parent modeling of exercise may be due to limitations in parental bandwidth due to competing demands, high levels of stress, or lack of access to consistent spaces for exercise. There is a lack of literature on housing insecurity and parenting behaviors with which our results can be compared. However, we know that housing instability is negatively associated with the health of both caregivers (15, 18) and children (15, 16). These associations could potentially be mediated through nutrition and physical activity-related behaviors of caregivers.

The study sample was predominantly Hispanic and low-income and included only parents of children with overweight or obesity, which provides new insight into parenting behaviors in this population; however, these associations may not generalize to other populations. No causal conclusions can be made from this analysis given the cross-sectional design. Due to concern of respondent burden with lengthy study measures, outcome measures were limited to individual items drawn from larger validated measures, which may limit validity. Additionally, the ACTS-MG was validated with African American and non-Hispanic white parents (13) and the CFPQ was validated with predominantly Caucasian parents with a higher median income and children of slightly younger age (4 to 6 years of age) relative to our sample (14), so the validity of the measures in our sample of predominantly Hispanic parents of school-aged children is unknown. Since exposure and outcome measures were self-reported, there is the potential for social desirability bias. Particularly, with the outcome measures, parents were asked questions regarding healthy foods, which requires them to subjectively report whether food is healthy and results in more variability compared to directly measuring the dietary quality of foods kept in the house and consumed by parents. While we did not use the comprehensive 18-item United States Department of Agriculture (USDA) food insecurity measure which may identify additional dimensions of food insecurity, the 2-item Hunger Vital Sign is a widely used measure that assesses for food insecurity risk and has been validated across multiple populations including caregivers of young children (11), youth and adolescents (19), and adults (20). There is no gold standard measure to screen for housing insecurity and existing screening tools capture different dimensions of housing-related risk (21). The 2-item housing insecurity measure used in this analysis primarily assessed for housing affordability and did not assess for other dimensions such as overcrowding, multiple moves, or homelessness, which may have different associations with the outcomes. We considered the possibility of examining the co-occurrence of food and housing insecurity by structuring the exposure as 4 mutually exclusive groups that incorporate both food and housing into one variable, however we were unable to perform this analysis due to limitations in sample size. Lastly, the household income variable had a large number of missing responses (*n* = 89), which were thought to be missing not at random (likely higher rates of missing for lower income levels), which resulted in the selection of a complete case analysis approach.

These findings demonstrate that food and housing insecurity may be barriers to parents engaging in healthy lifestyle parenting behaviors related to physical activity and nutrition. As accountable care organizations move towards screening for unmet social needs, such as food and housing insecurity, clinicians and pediatric weight management interventions should address these needs as important components of healthy lifestyle change. Additionally, disparities in unmet social needs and child health outcomes, including obesity, may be impacted by historical inequities and systemic racism (22, 23). Upstream multi-level interventions including public policy to address food and housing security should be implemented with an eye towards decreasing disparities and improving health outcomes, particularly in low-income communities and communities of color.

## Data Availability

The data analyzed in this study is subject to the following licenses/restrictions: The data presented in this study are available on request. Requests to access these datasets should be directed to kelsey.egan@bmc.org.
